# Sensitivity of Sinus Radiography Compared to Computed Tomogram: A Descriptive Cross-sectional Study from Western Region of Nepal

**DOI:** 10.31729/jnma.4824

**Published:** 2020-04-30

**Authors:** Manish Kiran Shrestha, Dilasma Ghartimagar, Arnab Ghosh, Adarsh Kumar Jhunjhunwala

**Affiliations:** 1 Department of Radiology, Gandaki Medical College Teaching Hospital, Pokhara, Nepal; 2 Department of Pathology, Manipal College of Medical Science, Pokhara, Nepal

**Keywords:** *computed tomography*, *maxillary*, *radiography*, *sinusitis*

## Abstract

**Introduction:**

Radiography of the paranasal sinuses is commonly used diagnostic modality. However, the trustworthiness of plain radiographic findings of paranasal sinuses is debatable. The intention of this study was to weigh the diagnostic soundness of plain radiograph of the paranasal sinuses to that of computed tomogram scan.

**Methods:**

This is a descriptive cross sectional study carried out in 110 participants in Department of Radiology of Gandaki Medical College from November 2017 to April 2018. Ethical approval is obtained from Institution review board (Ref. No.39/074/075). Sample size was calculated taking confidence level of 95%, expected prevalence of 14% and precision of 6.5% in population of 492098 in Province 4 of Nepal. Random sampling method was used. Data was enter in Statistical Package for the Social Sciences version 17 software and analysed.

**Results:**

A total of 110 participants are included in this study of which 62 (56.4%) are females and 48 (43.6%) are males with an overall mean age of 34.5 years. The commonly involved sinus was maxillary 56 (50.9%) followed by ethmoid 33 (30%) sinus. The overall sensitivity and specificity of detecting sinusitis by sinus radiography is higher for maxillary sinus (89.7% and 87%) followed by ethmoid (69.7% and 96.1%) and frontal (61.5% and 96.9%) sinuses.

**Conclusions:**

Sinus radiography is more sensitive for detecting pathologies in maxillary sinuses, while it is moderate for frontal, ethmoid sinuses and least for sphenoid sinuses. Diagnostic accuracy of computed tomogram scan is more, hence should be recommended to characterize the complex pathology and anatomy of the osteomeatal complex.

## INTRODUCTION

Radiography of the paranasal sinuses (PNS) is one of the commonly used diagnostic modality in assessment of the sinuses in this part of the country. Clinicians recommend plain radiograph to consolidate the clinical diagnosis, in suspicious cases and to plan further intervention or investigations such as computed tomogram (CT) scan or nasal endoscopy.^1,2^

Radiography of the PNS is a widely popular as the investigation is easily available, simple and affordable to the general public.^1–3^ Furthermore, most of the clinicians prefer conventional radiographs due to lower radiation dose compared to computed tomography (CT) scan, and faster scan times.^4^ However, the trustworthiness of plain radiographic findings of PNS is debatable and even an experienced radiologist may have troubles in reading PNS radiographs.^1–5^

The aim of this study was to weigh the diagnostic soundness of plain radiograph of the paranasal sinuses to that of the CT of the osteomeatal complex.

## METHODS

This was a descriptive cross sectional study carried out in 110 participants in Gandaki Medical College for a period of 6 months from November 2017 to April 2018. Taking confidence level of 95%, expected prevalence of 14% in population of 492098 in Province 4 of Nepal and precision of 6.5% the sample size is calculated using the formula;

n= Z^2^ × p × q / e^2^

Where,

n = sample size
Z = 1.96 at 95% CI
p = prevalence, 14%
q = 1 - p
e = Margin of error, 6.5%
n = Z^2^ × p × q / e^2^
n = (1.96)^2^ × (0.14 × 0.86) / (0.065)^2^
n = 110

Random sampling method was used. All the data were gathered and calculations performed using SPSS version 17. The participants selection criteria includes clinical suspicion for sinusitis, recurrent sinusitis, ambiguous cases, patients not responding to treatment or participants with dubious PNS radiographs. Participants with a PNS radiograph prior to CT were selected. Patients who underwent only CT scan or radiography only or both the scans more than 14 days apart were not included in the study.

Water's view was taken for sinus radiography. The participant laid down prone; the patient's orbito-meatal line was angled to 37 degrees to the table bucky surface/ image receptor (IR). The central ray (CR) was aligned perpendicular to the IR to exit at the acanthion. Source image distance (SID) was set to 100cm (40inches). Open mouth Water's view was taken to picture the ethmoid and sphenoid sinuses as well.

X-rays were performed on K-RAM GE machine 300. Exposure factor 75KVp 200mAs. CT examination was carried out with 4 slice CT scanner (Toshiba Asteon). The participant was made to lie in prone position with the neck hyperextended, in coronal plane with image interval of 3mm through the osteomeatal complex and 5mm in posterior sections. The tube potential was 150kVP and tube current 150mA. The images were viewed at WL of 2200-2700 and WW of 350-400.

The PNS radiographs and CT scans were reviewed separately by the radiologists. The criteria used to define sinusitis on PNS radiograph was total opacification, air fluid level and mucoperiosteal thickening of 3mm or greater.^1,2^

## RESULTS

A total of 110 participants were included in this study of which 62 (56.4%) were females and 48 (43.6%) were males. The age ranged from 10yrs to 67yrs old (Table 1).

**Table 1:** Age wise distribution of cases.

Age group	Male	Female	No of cases n (%)
< 10yrs	1	-	1 (0.9)
11-20	7	11	18 (16.36)
21-30	12	20	32 (29.09)
31-40	11	15	26 (23.63)
41-50	8	5	13 (11.81)
51-60	8	6	14 (12.72)
61-70	1	5	6 (05.45)
Total	48	62	110 (100)

Overall mean age was 34.5 years with standard deviation of 14.5 and the median age was 32 years. The prevalent gender was females.

The maximum involvement of the sinus showed by CT scan was maxillary sinus 56 (50.9%) cases followed by ethmoid sinus 33 (30%) cases, sphenoid sinus 17 (15.4%) cases and frontal sinus 13 (11.8%) cases. Only in one case there was no involvement of any of the sinuses (Table 2).

**Table 2:** Frequency of sinus involvement by CT scan.

Sinuses	Numbers n
Frontal	13 (11)
Ethmoid	33 (30)
Maxillary	56 (50.9)
Sphenoid	17 (15.5)
None	1 (0.9)

In this study, the sensitivity and specificity of detecting sinusitis by sinus radiography was maxillary sinus with 89.2% and 87%. This was followed by ethmoid sinus with sensitivity and specificity of 69.7% and 96.1% and frontal sinus with sensitivity and specificity of 61.5% and 96.9%. The overall sensitivity and specificity of detecting sinusitis by sinus radiography was higher for maxillary sinus. The accuracy for frontal and sphenoid sinus was 92.7% and 90.9% respectively. The disease prevalance for maxillary sinus, ethmoid sinus, sphenoid sinus and frontal sinuses were 50.9%, 30%, 15.4% and 11.8% respectively (Table 3).

**Table 3:** Sensitivity and specificity of PNS radiograph compared to CT.

Sinuses	Sensitivity %	Specificity %	Accuracy %	Disease prevalence %
Frontal	61.5	96.9	92.7	11.8
Ethmoid	69.7	96.1	88.1	30
Maxillary	89.2	87	88.1	50.9
Sphenoid	41.1	100	90.9	15.4

Hypoplastic frontal sinuses were seen in 24.5%. Deviated nasal septum (DNS) to the right side was seen in 34.5% and DNS to the left was seen in 33.6%

## DISCUSSION

The study showed overall sensitivity and specificity of detecting sinusitis by sinus radiography was higher for maxillary sinus (89.7% and 87%) followed by ethmoid (69.7% and 96.1%) and frontal (61.5% and 96.9%) sinuses. The commonly involved sinus was maxillary (50.9%) followed by ethmoid (30%) sinus.

The endemic age group in our observation was 34.5 years with the female genders being more common. The prevalent gender presenting with sinus pathologies, in study done by Hussein AO et al were women (53.7%) while commonly involved age group was 19-29 years of age.^6^ In investigation by Burke et al the predominant gender was females (73.3%) with mean age of 37 years.^1^ In a study done by Chiu PY et al the average age involved was 52.3 years.^3^ The stimulus of which were attributed to upper respiratory tract infections, environmental factors and geographical location. Maxillary sinus was the most frequently involved sinus (72%) followed by ethmoid (45.4%), frontal (31.7%) and sphenoid (27.2%) sinuses.^6^ Maduforo CO et al also published similar report stating maxillary sinus to be the predominant sinus involved (66.7%).^7^ In the observation made by Chiu PY et al, the overall sensitivity of detecting sinusitis was 92% with higher sensitivity in maxillary (88.6%) frontal (88.9%) sinuses and lower in ethmoid (57.1%) and sphenoid (28.6%) sinuses.^3^

Konen E et al published the result of their study on 134 patients where the sensitivity of abnormality in maxillary sinus was 67.7%, specificity 87.6% and accuracy 78.6%.^8^ The sensitivity for diagnosing any disease in the frontal and ethmoid sinuses varied widely between different observers ranging from 1.9-54.0% and 0-58.9%, respectively while the sensitivity for the sphenoid sinus was very low ranging from 0-3.8%.^8^ In our study, the sensitivity for detection of sinusitis on PNS radiograph was highest for maxillary sinus (89.2%), followed by ethmoid(69.7%) and frontal sinus (61.5%) while it was least for sphenoid sinus (41.1%). In study by Aalokken TM et al. the specificity is high for all sinuses but sensitivity is relatively low, except for maxillary sinus where the sensitivity is 80% followed by ethmoid (41%), frontal (39%) and sphenoid (25%).^9^ The overall plain film sensitivity for detecting sinusitis in reports by Burke et al ranged from 48% to 67% but for maxillary sinus it was higher with sensitivity of 70% and specificity of 96-100%.^1^

These surveys exhibit that plain radiograph has considerable sensitivity and specificity for maxillary sinus but the sensitivity was lower for other sinuses and least for sphenoid sinuses. Apart from that, the false negative cases were higher compared to false positive cases in our review. Radiograph of paranasal sinuses was dependent on operator's expertise as well as the participants. Improper radiographic techniques such as exposure factors, positioning of the tube or participants head as well as the complex anatomy of the sinuses can all influence the presentation of the radiographs.^3,6,10^ Furthermore there can be variations in interpretation of radiographs by different observers as was seen in the study done by Burke et al.^1^ In a comparative study of radiological and antroscopic findings of sinuses done by Gupta SC et al the correlation between X-ray and antroscopic diagnosis is 52.4% while with CT it is 90%.^10^

The limitation to our study was the time delay between the x-ray to the CT scans of the paranasal sinuses, which varied from 4 to 10 days. Longer gap was not favorable as there may be resolution or on the contrary increase of the sinus pathology and can influence the end result. Endoscopic evaluation of the sinuses coupled with culture from the involved sinuses could have supplemented the research.

Both x-ray and CT has radiation involved with it. The mean effective dose in radiograph of paranasal sinus is 0.0398 mSv.^11^ The radiation dose is 0.047 mSv in men, 0.051 mSv in women in low dose CT; 0.70 mSv in men and 0.76 mSv in women in standard dose MDCT (150 mAs); which was lower than the annual dose from natural radiation (1 mSv).^9,12^ In an effort to reduce radiation dose it is more appropriate to utilize nasal endoscopy when available. Low dose CT of the sinuses should be used judiciously to delineate the complex anatomy of the sinuses and review the extent of sinus pathology or bony erosion on clinical suspicion.^13^

Radiograph of the paranasal sinuses should only be recommended if it can offer additional information to the clinician.

## CONCLUSIONS

Sinus radiography was more sensitive for detecting pathologies in maxillary sinuses, while it was moderate for frontal, ethmoid sinuses and least for sphenoid sinuses. Diagnostic accuracy of CT scan was more than plain radiography, hence should be recommended to characterize the complex pathology and anatomy of the osteomeatal complex.

## Conflict of Interest

**None.**
